# Fast accurate missing SNP genotype local imputation

**DOI:** 10.1186/1756-0500-5-404

**Published:** 2012-08-03

**Authors:** Yining Wang, Zhipeng Cai, Paul Stothard, Steve Moore, Randy Goebel, Lusheng Wang, Guohui Lin

**Affiliations:** 1 Department of Computing Science, University of Alberta, Edmonton, Alberta T6G 2E8, Canada; 2 Department of Computer Science, Georgia State University, Atlanta, GA 30303, USA; 3 Department of Agriculture, Food, and Nutritional Science, University of Alberta, Edmonton, Alberta T6G 2C8, Canada; 4 Department of Computer Science, City University of Hong Kong, Kowloon, Hong Kong SAR, China

## Abstract

**Background:**

Single nucleotide polymorphism (SNP) genotyping assays normally give rise to certain percents of no-calls; the problem becomes severe when the target organisms, such as cattle, do not have a high resolution genomic sequence. Missing SNP genotypes, when related to target traits, would confound downstream data analyses such as genome-wide association studies (GWAS). Existing methods for recovering the missing values are successful to some extent – either accurate but not fast enough or fast but not accurate enough.

**Results:**

To a target missing genotype, we take only the SNP loci within a genetic distance vicinity and only the samples within a similarity vicinity into our local imputation process. For missing genotype imputation, the comparative performance evaluations through extensive simulation studies using real human and cattle genotype datasets demonstrated that our nearest neighbor based local imputation method was one of the most efficient methods, and outperformed existing methods except the time-consuming fastPHASE; for missing haplotype allele imputation, the comparative performance evaluations using real mouse haplotype datasets demonstrated that our method was not only one of the most efficient methods, but also one of the most accurate methods.

**Conclusions:**

Given that fastPHASE requires a long imputation time on medium to high density datasets, and that our nearest neighbor based local imputation method only performed slightly worse, yet better than all other methods, one might want to adopt our method as an alternative missing SNP genotype or missing haplotype allele imputation method.

## Background

Genetic fine-mapping for complex traits (such as human cancers, diabetes, mental illness, cattle milk products, beef quality, etc.) is still a great challenge for geneticists. With the availability of millions of *single nucleotide polymorphisms* (SNPs), research sees new potentials via using these common variants — the *genome-wide association studies* (GWAS) [1]. In general, GWAS, either case-control or categorical or quantitative, requires many samples along with large and dense SNP marker sets, which are apt to contain a significant number of missing data. For diploid species such as human and cattle, high density SNP microarray chips can give an unphased genotype value for each SNP marker. For humans with a superb resolution reference genome, the current general purpose high-density gene chips are estimated to contain a portion of missing genotypes and genotyping errors in the range from 0.05% to 5%; for other species such as cattle, their whole genomes have not yet been sequenced ideally, and consequently their gene chips could contain many more missing data and errors, up to 20% [2,3].

The unphased genotype data is certainly the No. 1 issue that complicates GWAS. The missing genotypes present in the dataset, particularly when the percentage is high, also challenge the association study methods [4-9]. When markers with missing genotypes are recognized extremely suspecting, one can choose to repeat the genotyping or modify the data analysis tools to accommodate the missing data. However, both approaches are expensive in terms of labor and cost. Alternatively, one can try to computationally infer the missing data, otherwise known as *imputation*, at a minimal labor and cost. Nevertheless, it should be pointed out that when the genotype data are generated by low-to-medium density arrays, imputation might not be accurate and thus should be avoided, as otherwise the imputed data would confound the gene-trait association analyses [3,4,10-19].

In this paper, we propose the *local* imputation approach that has several advantages over existing global imputation methods, where our computational inference is done based on the following biological observations. For our target diploid organisms human, mouse and cattle, chromosomes come in pairs. The Mendelian law of inheritance states that, for each individual, one of a pair of homologous autosomes is inherited from her father (the paternal) and the other from her mother (the maternal). In general, a child does not inherit a complete parental chromosome from each parent, as *recombination* (or *crossover*) events occur. That is, during the meiosis process where the two parental chromosomes get duplicated and shuffled and four chromatids are made, one of the chromatids is passed on to the child. Between any two consecutive SNP loci along the chromosome, the recombination rate is described by the *genetic distance* between them. The human genetic map can be obtained from the HapMap [20].

For each individual, the two alleles at a SNP locus of a pair of homologous chromosomes together is called the *genotype* of this locus. The genotype of a locus does not specify which allele comes from which one of the two chromosomes. Thus, the genotype of a locus can be denoted as an unordered pair of two alleles, and the genotype of a pair of homologous chromosomes can be viewed as a sequence of unordered pairs of SNP alleles. A SNP locus is *homozygous* if its two alleles are the same, or *heterozygous* otherwise. On the contrary, the *haplotype* of a SNP locus specifies the parental origin for the two alleles, and a chromosomal haplotype consists of all the alleles, one for each SNP locus, of the same parental origin.

From the Mendelian law of inheritance and the fact that recombination is a rare event, we know that for each short haplotype allele (over a short chromosomal region), there are likely many individuals share it due to *identical-by-descent* (IBD). The length of such a haplotype allele could vary from location to location, and it is likely population dependent. In fact, this *coalescent* theory underlies many existing *haplotyping*-based imputation methods using a variety of techniques, such as hidden Markov models (HMMs) and the expectation maximization (EM) algorithm [8]. Among them, the most promising and the most applicable methods to GWAS analysis include fastPHASE [17], MaCH [21], and Impute [6] that are based on Li and Stephens “product of approximate conditionals” framework [22], and Beagle [5] that is based on Browning “localized haplotype clustering” model [23]; while many others are either out of date or too computationally intensive [3,10-12,14,16].

Despite the fact that several of these haplotyping-based imputation methods can bypass the haplotyping phase, they impute the missing genotypes to satisfy various haplotyping needs, and thus their imputation accuracies highly depend on the haplotyping assumptions [8]. It is worth mentioning that more problematically sometimes, the haplotyping-based imputation methods may alter a certain percentage of known genotype as well to satisfy their haplotyping needs [17], making the task of missing genotype imputation difficult to evaluate.

There are also several imputation methods that are not based on haplotyping, but indirectly use the coalescent theory to impute missing genotypes without considering haplotype phases [18,19,24]. Their representatives include regression methods and *k*-nearest neighbor methods. The recent one nearest neighbor method Npute [18], extended to KnnWinOpti and SnpShuttle [24], proposes to use surrounding SNP loci to compute the genotype similarity between two samples, and imputes the target missing genotype with the genotype from the nearest sample. Note that the current code of Npute does not accept heterozygosities in the datasets, and thus it is essentially for missing haplotype allele imputation [25].

In this paper, we focus on direct missing genotype imputation, to avoid the ambiguous genome block partitioning, the time-consuming haplotyping stage, and the haplotyping uncertainties in the haplotyping-based imputation methods [3,8,16,17]. To this end, we present a new nearest neighbor method denoted as NN and a weighted variant denoted as WNN. Both methods follow the coalescent theory that the target individual has a genotype sequence similar to one from the population; they also respect the observation that recombination events are rare to infer the target missing value using only nearby SNP loci, where the vicinity is defined by a genetic distance (or recombination rate) threshold; while NN treats these nearby SNP loci equally, WNN method steps further to weight these SNP loci by the reciprocal of their genetic distance to the target locus. Therefore, both NN and WNN are *local* imputation methods. Besides these two methods, we have also implemented several other local machine learning imputation methods, including a local support vector machine with a radial basis function kernel (SVM), a local neural network (NeuralNet), and a local first order Markov chain (MC).

We have used a (Southwest African) human SNP genotype dataset downloaded from the HapMap [20], an inbreed mouse SNP genotype dataset extracted from the NIEHS/Perlegen resequencing project, a medium density and a high density cattle SNP genotype datasets from our Beef Cattle Whole Genomic Selection projects, for extensive comparative studies between our methods and two existing best methods fastPHASE [17] and Npute [18] through simulation experiments on various settings. The results show that our NN and WNN methods were among the most efficient methods, and outperformed existing methods except fastPHASE in missing SNP genotype imputation; furthermore, on missing SNP haplotype allele imputation, our methods performed comparably again fastPHASE, sometimes even better. Note that the original human and mouse datasets all contain a certain percentage of missing values. The imputed datasets by our NN method, the simulated datasets, all our implemented methods in Java, and the detailed imputation result statistics are available in the supplementary materials [26].

## Results

### Datasets

To validate our NN and WNN methods, and to compare against the other three machine learning imputation methods SVM, NeuralNet and MC that we have implemented and two existing best imputation methods fastPHASE and Npute, we used four real SNP genotype datasets for simulation studies. The first dataset is a human population SNP genotype dataset for chromosome 17 obtained from the International HapMap project (Phase I) [20] (file name “ http://genotypes_chr17_ASW_r27_nr.b36_fwd.txt.gz”), which contains 40,775 SNPs and 83 individuals of African ancestry in Southwest USA (ASW). This original dataset contains 0.268% missing calls. For simulation study purpose, SNPs with missing values are removed and the dataset is left with only 34,071 (or 83.60%) SNP markers. This final dataset is simply called the human dataset in the sequel. The corresponding genetic map for human chromosome 17 was also downloaded from the HapMap (file name “ http://genetic_map_chr17_b36.txt”). The second dataset was extracted from the NIEHS/Perlegen resequencing project, which provides a high-resolution map of 16 common mouse strains. We used again the chromosome 17 SNP whole genome dataset, which is made up of 15 inbred mouse strains genotyped at 288,229 SNP loci along the chromosome, and with 11.1% missing calls. Our examination confirmed that all the genotype values are homozygous, and thus the dataset can be used for simulation studies on missing SNP haplotype allele imputation. To this purpose, we removed those SNP loci containing missing values; the final dataset is left with 144,820 (or 50.24%) SNP markers, which is referred to as the mouse dataset in the sequel.

The human and the mouse datasets are of very high density, and they are referred to as density-1 datasets. From these two density-1 datasets, we respectively retain every tenth and every hundredth SNP markers to simulate medium and low density datasets, referred to as density-0.1 and density-0.01 datasets. Namely, the density-0.1 and density-0.01 human datasets contain 3,408 and 341 SNP markers respectively; the density-0.1 and density-0.01 mouse datasets contain 14,482 and 1,449 SNP markers respectively.

Our target datasets are for cattle, which were obtained from the Beef Cattle Whole Genomic Selection projects (more datasets are expected to come in the near future). One such dataset has a medium density of 51,828 markers and 469 genotyped animals, and the other has a higher density of around 700K markers and 64 animals. The animals in both datasets are considered largely unrelated, or at least very distantly related. These two datasets contain 0.078% and 2.224% missing genotypes, respectively. For chromosome 17, the low density dataset contains 1,508 SNP markers, 237 of them have missing values; the high density dataset contains 22,266 SNP markers, but unfortunately only 5,487 of them have genotype values from all 64 animals. We refer to these two cattle datasets on chromosome 17 in the sequel as the low density (ld) dataset and the high density (hd) dataset, respectively. All the four datasets are summarized in Table 1.

**Table 1:** Dataset summary

**Dataset:**	**human**	**mouse**	**hd cattle**	**ld cattle**
Physical length	79 mbp	95 mbp	76 mbp	76 mbp
#samples	83	15	64	469
#SNPs	40,755	288,229	22,266	1,508
Missing genotype rate	0.268%	11.1%	2.224%	0.078%
#complete SNPs	34,071	144,820	5,487	1,271

The summary of the four datasets we used in the simulation studies. These four datasets are all on chromosome 17, for human, mouse and cattle. The “physical length” refers to the chromosome length, in million basepairs (mbp). “#samples” is the number of individuals genotyped in the dataset, “#SNPs” is the number of SNP markers in the original dataset, and “missing genotype rate” refers to the percentage of missing genotype values in the original dataset. “#complete SNPs” is the number of SNP markers at which all samples have genotype values. All the other SNP markers were removed, leaving a complete sub-dataset to be used in the simulation studies.

On each of the above three human, three mouse, and two cattle datasets, we uniformly randomly mask 0.5%, 1%, 2%, 5%, 10% and 20% genotypes, respectively, to mimic different missing rates due to various reasons. At each missing rate, a total of 10 simulated SNP genotype datasets are *independentlyidentically* generated; every imputation method, NN, WNN, SVM, NeuralNet, MC, fastPHASE, Npute (when applicable), and BaseLine is applied on them and the average imputation accuracy, which is the number of correctly imputed genotypes over the total number of missing genotypes, is reported as the method performance at this missing rate. Here BaseLine is a simple majority voting using the most frequent value at the target SNP locus from the samples with a known genotype value.

### Imputation accuracy comparison

In the sequel, we use a triplet to denote one kind of datasets; for example, 0.1-human-2% denotes the density-0.1 human datasets with missing rate 2%. We run fastPHASE and Npute strictly following their instructions, but note that Npute does not apply for human or cattle datasets due to heterozygosities. To run our implemented local imputation methods, we will need to set up genetic distance thresholds. We adopt a grid search scheme to use {0.01, 0.02, 0.03, 0.04, 0.05} / *density* centi-Morgans; nevertheless, instead of reporting the best performance we collect the statistics across all five thresholds. It is worth pointing out that, due to random masking, there could be some missing entries for which there are no neighboring SNP loci within the covering window and thus no information for inference, which are excluded from imputation accuracy calculation for fairness.

#### Missing genotype imputation

For each (human and cattle) triplet, we ran all imputation methods on the ten simulated datasets using all five associated different genetic distance thresholds. For fastPHASE and BaseLine, genetic distance threshold does not have any effects on their run; but their imputation accuracies could slightly vary with the threshold since some missing genotype values can be excluded from statistics (noted in the last paragraph).

**Figure 1:**
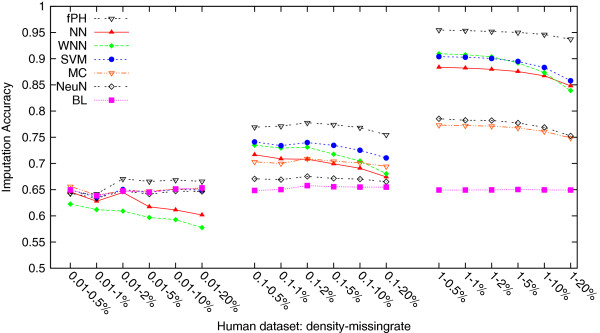
**Average imputation accuracies of the seven methods, fastPHASE (fPH), NN, WNN, SVM, MC, NeuralNet (NeuN) and BaseLine (BL), on the three different density level human datasets: density-0.01, density-0.1 and density-1.** The general tendencies are, on the low density datasets, no methods performed significantly better than BaseLine, which is a simple majority vote; on the median density datasets, methods started to perform better than BaseLine, with fastPHASE being the best and our methods NN and WNN and SVM performing not significantly different; on the high density datasets, the differences between fastPHASE and the second group, between the second and the third group of MC and NeuralNet, and between the third group and BaseLine became significantly larger.

For each human triplet, we calculated the average imputation accuracy for a method by taking the average over all five runs using different genetic distance thresholds on the 10 simulated datasets. That is, it is the average of 50 accuracies. Table 2 lists these average imputation accuracies on all three density human datasets each with six missing rates, one column for a method. They are also plotted in Figure 1 for easier view of performance difference. As one sees, the general tendencies are 1) on the low density datasets, no methods performed significantly better than BaseLine; 2) on the median density datasets, methods started to perform better than BaseLine, with fastPHASE being the best (*p*-values < 0.0001) and our methods NN and WNN and SVM performing not significantly different; 3) on the high density datasets, the differences between fastPHASE and the second group, between the second and the third group of MC and NeuralNet, and between the third group and BaseLine became significantly larger. It is worth pointing out that our methods NN and WNN ran in seconds to minutes on a dataset, while fastPHASE ran thousands of times slower, in days. More detailed results on runtime statistics are in the next subsection “Imputation time comparison”.

**Table 2:** Average imputation accuracies on the three density human datasets

**Methods**	**fPH**	**NN**	**WNN**	**SVM**	**NeuN**	**MC**	**BL**
0.01-0.5%	.6427	.6456	.6225	.6499	.6431	**.6561**	.6495
0.01-1%	**.6418**	.6279	.6119	.6353	.6338	.6399	.6384
0.01-2%	**.6707**	.6447	.6091	.6503	.6474	.6474	.6477
0.01-5%	**.6656**	.6171	.5968	.6452	.6415	.6474	.6449
0.01-10%	**.6683**	.6113	.5927	.6506	.6472	.6510	.6513
0.01-20%	**.6658**	.6016	.5776	.6492	.6465	.6515	.6536
0.1-0.5%	**.7692**	.7167	.7348	.7412	.6707	.7032	.6484
0.1-1%	**.7712**	.7087	.7294	.7340	.6691	.6999	.6501
0.1-2%	**.7778**	.7081	.7311	.7399	.6753	.7092	.6577
0.1-5%	**.7741**	.6993	.7176	.7345	.6714	.7043	.6556
0.1-10%	**.7684**	.6908	.7048	.7252	.6701	.7011	.6549
0.1-20%	**.7547**	.6742	.6804	.7105	.6652	.6944	.6548
1-0.5%	**.9548**	.8836	.9094	.9036	.7854	.7732	.6493
1-1%	**.9537**	.8822	.9077	.9028	.7826	.7722	.6493
1-2%	**.9520**	.8796	.9036	.9006	.7820	.7713	.6495
1-5%	**.9502**	.8755	.8919	.8948	.7774	.7679	.6503
1-10%	**.9462**	.8673	.8736	.8833	.7689	.7611	.6494
1-20%	**.9373**	.8481	.8391	.8579	.7526	.7488	.6493

Average imputation accuracies on the three density human datasets. At each missing rate, the highest accuracy is in bold. ‘fPH, NeuN’ stand for ‘fastPHASE, NeuralNet’, respectively.

**Figure 2:**
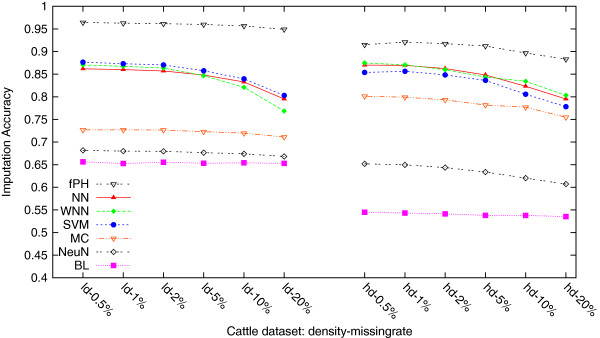
**Average imputation accuracies of the seven methods, fastPHASE (fPH), NN, WNN, SVM, MC, NeuralNet (NeuN) and BaseLine (BL), on the two real cattle datasets.** fastPHASE performed statistically significantly better (*p*-values < 0.0001) than all the other six methods on both datasets; our methods NN and WNN and SVM performed no differently, and significantly better (*p*-values < 0.0001) than MC, NeuralNet and BaseLine. Interestingly, it is challenging to argue which one of the two datasets is easier to impute. As far as we know, the low density dataset has been curated multiple times when it was used in GWAS, and it contains much more samples (469) which are certainly helpful for imputation; the high density dataset was more recently generated, has not been used in GWAS, and it contains only 64 samples. Nevertheless, the performance differences between fastPHASE and the second group of NN, WNN and SVM, and between the second group and the other three methods are similar to those on high density human datasets.

For each cattle triplet, we also calculated the average imputation accuracy for a method by taking the average over all five runs using different genetic distance thresholds on the 10 simulated datasets. Table 3 and Figure 2 list and plot, respectively, these average imputation accuracies on these two density cattle datasets each with six missing rates. As one sees, 1) fastPHASE once again performed statistically significantly better (*p*-values < 0.0001) than all the other six methods; 2) our methods NN and WNN and SVM performed no differently, and significantly better (*p*-values < 0.0001) than MC, NeuralNet and BaseLine. Interestingly, it is challenging to argue which one of the two datasets is easier to impute. As far as we know, the low density dataset has been curated multiple times when it was used in GWAS, and it contains much more samples (469) which are certainly helpful for imputation; the high density dataset was more recently generated, has not been used in GWAS, and it contains only 64 samples. Nevertheless, the performance differences between fastPHASE and the second group of NN, WNN and SVM, and between the second group and the other three methods are similar to those on high density human datasets. Lastly, it is again worth pointing out that our methods NN and WNN ran in seconds to minutes on a dataset, while fastPHASE and SVM ran thousands of times slower, in hours to days (see “Imputation time comparison”).

**Table 3:** Average imputation accuracies on the two cattle datasets

**Methods:**	**fPH**	**NN**	**WNN**	**SVM**	**NeuN**	**MC**	**BL**
hd-0.5%	.9151	.8701	.8748	.8538	.6518	.8012	.5449
hd-1%	.9209	.8695	.8707	.8563	.6495	.7991	.5432
hd-2%	.9174	.8624	.8599	.8484	.6437	.7933	.5412
hd-5%	.9123	.8482	.8436	.8362	.6336	.7817	.5379
hd-10%	.8968	.8232	.8344	.8057	.6204	.7771	.5378
hd-20%	.8831	.7951	.8032	.7782	.6072	.7548	.5352
ld-0.5%	.9643	.8618	.8705	.8765	.6817	.7269	.6563
ld-1%	.9627	.8601	.8674	.8732	.6799	.7270	.6527
ld-2%	.9616	.8571	.8636	.8704	.6796	.7265	.6552
ld-5%	.9598	.8480	.8468	.8577	.6765	.7230	.6532
ld-10%	.9566	.8328	.8209	.8395	.6740	.7169	.6542
ld-20%	.9492	.7951	.7686	.8029	.6680	.7110	.6528

Average imputation accuracies on the two cattle datasets, the high-density one is 700K and the low-density one is 60K. At each missing rate, the highest accuracy is in bold. ‘fPH, NeuN’ stand for ‘fastPHASE, NeuralNet’, respectively.

#### Missing haplotype allele imputation

**Figure 3:**
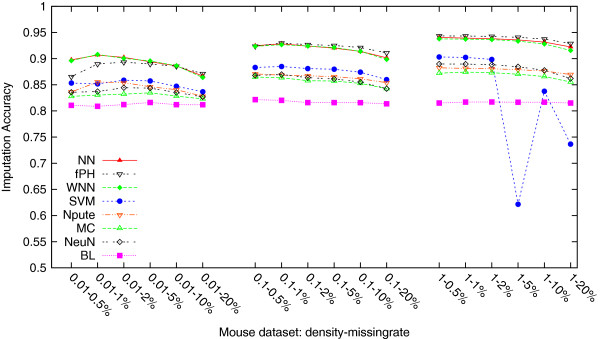
**Average imputation accuracies of the eight methods, fastPHASE (fPH), NN, WNN, SVM, Npute, MC, NeuralNet (NeuN) and BaseLine (BL), on the three different density level mouse datasets: density-0.01, density-0.1 and density-1.** Compared to the above missing genotype imputation, the general tendencies are different: 1) in all three density level datasets, our methods NN and WNN performed no differently from fastPHASE, and even slightly better on the density-0.01 datasets; all three of them performed statistically significantly better (by ∼6%, *p*-values <0.0001) than previously the best method Npute. 2) There are only three groups here, the first group consists of fastPHASE, NN and WNN; the second group includes SVM, Npute, MC and NeuralNet; and the last group contains only BaseLine. 3) Even on the low density datasets, all other methods performed better than BaseLine; on the median and high density datasets, the gaps became significantly larger. It should be pointed out that SVM performed very strangely on the high density datasets with missing rates 5% (and 10%, 20%). We have in fact separately tested multiple times, but the same pattern was always there.

For missing haplotype allele imputation comparison, we ran all imputation methods, including Npute this time as it is designed for such imputation [18], on the ten simulated mouse datasets using five different genetic distance thresholds. Again, for fastPHASE, Npute and BaseLine, genetic distance threshold does not have any effects on their run, but their imputation accuracies could vary due to some missing values being excluded from the statistics. Table 4 lists these average imputation accuracies on all three density mouse datasets each with six missing rates, and they are plotted in Figure 3 for easier view of performance difference. This time, 1) in all three density level datasets, our methods NN and WNN performed no differently from fastPHASE, and even slightly better on the density-0.01 datasets; all three of them performed statistically significantly better (by ∼6%, *p*-values < 0.0001) than previously the best method Npute. Compared to the above missing genotype imputation, the general tendancies are different: 2) there are only three groups here, the first group consists of fastPHASE, NN and WNN; the second group includes SVM, Npute, MC and NeuralNet; and the last group contains only BaseLine. 3) Even on the low density datasets, all other methods performed better than BaseLine; on the median and high density datasets, the gaps became significantly larger. It should be pointed out that SVM performed very strangely on the high density datasets with missing rates 5% (and 10%, 20%). We have in fact separately tested multiple times, but the same pattern was always there.

**Table 4:** Average imputation accuracies on the three density mouse datasets

**Methods:**	**Npute**	**NN**	**WNN**	**fPH**	**SVM**	**NeuN**	**MC**	**BL**
0.01-0.5%	.8363	**.8977**	.8960	.8655	.8534	.8357	.8278	.8106
0.01-1%	.8554	.9068	**.9079**	.8898	.8517	.8371	.8322	.8088
0.01-2%	.8542	**.9023**	.9006	.8932	.8588	.8445	.8322	.8121
0.01-5%	.8462	.8940	**.8955**	.8898	.8573	.8436	.8347	.8162
0.01-10%	.8406	.8861	**.8865**	.8854	.8470	.8354	.8283	.8120
0.01-20%	.8281	.8668	.8639	**.8712**	.8366	.8268	.8231	.8118
0.1-0.5%	.8708	**.9247**	.9236	.9237	.8831	.8680	.8647	.8215
0.1-1%	.8685	.9283	.9267	**.9296**	.8850	.8697	.8634	.8203
0.1-2%	.8672	.9241	.9240	**.9269**	.8810	.8636	.8578	.8159
0.1-5%	.8655	.9201	.9212	**.9252**	.8796	.8611	.8571	.8160
0.1-10%	.8617	.9140	.9139	**.9212**	.8741	.8553	.8527	.8158
0.1-20%	.8541	.9015	.8986	**.9112**	.8598	.8426	.8426	.8136
1-0.5%	.8825	.9405	.9377	**.9434**	.9032	.8898	.8723	.8152
1-1%	.8814	.9392	.9373	**.9432**	.9023	.8896	.8742	.8169
1-2%	.8806	.9381	.9364	**.9426**	.8986	.8885	.8730	.8171
1-5%	.8788	.9358	.9334	**.9408**	.6215	.8845	.8703	.8166
1-10%	.8763	.9317	.9280	**.9375**	.8377	.8777	.8657	.8167
1-20%	.8695	.9223	.9156	**.9290**	.7365	.8621	.8553	.8151

Average imputation accuracies on the three density mouse datasets. At each missing rate, the highest accuracy is in bold. ‘fPH, NeuN’ stand for ‘fastPHASE, NeuralNet’, respectively.

### Imputation time comparison

All our experiments were run on a CPU cluster, where each node consists of a dual AMD Opteron 2350 quad core with 64-bit CPU’s. The CPU’s run at 2.0GHz, have an 800 MHz HyperTransport bus, with a primary cache of 64KB I + 64KB D per core, a secondary cache of 512 KB I+D per core, and a 2MB L3 cache per chip. In general, our methods NN and WNN, and MC and BaseLine were very fast, seconds per dataset; (Npute and) NeueralNet needed some more time to impute a dataset, though not too long; fastPHASE was always the slowest, and could take more than a week on a high-density dataset. For instance, for imputing the original human dataset (40,775 SNPs, 83 ASW individuals, 0.268% missing calls), fastPHASE took about eight days while our NN took less than three seconds. SVM appeared to be the second slowest, likely due to its internal training.

Table 5 lists the detailed average runtime of all eight methods on four datasets at missing rate 0.5%: namely 0.01-human-0.5%, 0.01-mouse-0.5%, hd-cattle-0.5%, ld-cattle-0.5%. The average is taken over the 10 simulated datasets associated with the triplet, and 5 genetic distance thresholds except fastPHASE, Npute and BaseLine. One can see from this table that, in general, fastPHASE is thousands of times slower than our methods NN and WNN, while from Tables 2, 3, and 4 that the imputation accuracy of our methods are very close to that of fastPHASE. This supports the adoption of our NN and WNN as alternative imputation methods for fastPHASE.

**Table 5:** Average imputation runtime on four datasets, in seconds

**Methods:**	**fPH**	**NN**	**WNN**	**SVM**	**NeuN**	**MC**	**BL**	**Npute**
0.01-human-0.5%	860.44	0.22	0.28	156.09	13.08	0.10	0.07	-
0.01-mouse-0.5%	530.59	0.17	0.16	33.47	0.43	0.12	0.08	21.08
hd-cattle-0.5%	6178.95	0.37	0.41	931.16	43.79	0.22	0.16	-
ld-cattle-0.5%	36209.82	1.15	2.41	22570.29	847.00	0.54	0.18	-

Average imputation runtime of all methods on four datasets: 0.01-human-0.5%, 0.01-mouse-0.5%, hd-cattle-0.5%, ld-cattle-0.5%, where the average is taken over 10 simulated datasets (with 5 runs using different genetic distance thresholds, except fastPHASE, Npute, BaseLine). ‘fPH, NeuN’ stand for ‘fastPHASE, NeuralNet’, respectively. Note that Npute spent most of its time, 20.62 out of the 21.08 seconds, in training for selecting the best window size in the range [1,50].

## Discussion

### The limit of imputation

On all of our simulated datasets, 180 human, 180 mouse and 120 cattle, we have never seen a 100% imputation accuracy by any method, using any covering window size (if applicable). Besides the limitation of methodologies, the small sample size in the datasets could be another cause. Currently we do not have quantified effects of the sample size, for the reason that all human, mouse and high-density cattle datasets have an already small number of samples, while the 469-cattle dataset is of only medium density and thus not suitable for simulation studies.

### The effects of density

From Tables 2 and 4, one can see that from low to medium to high densities, the imputation accuracies of most imputation methods increase. We believe this is reasonable since a higher density provides more neighboring SNPs, and consequently greater linakge disequilibrium, for imputation purpose. Interestingly, on cattle datasets, it is challenging to claim that the high density one is easier to impute. From Table 3 and Figure 2, one can see that only NN, WNN, SVM and MC performed better on the high density datasets, while fastPHASE, NeuralNet and BaseLine all performed worse. We suspect that the much larger sample size (*i.e.* 469) in the low density dataset could help fastPHASE for imputation, as it assumes a certain number of haplotype allele clusters. This suggests that when the sample size is smaller, our methods NN and WNN could be more suitable imputation methods.

Since fastPHASE takes more advantage of coalescent theory to perform haplotyping and imputation, it is expected to outperform our methods NN and WNN, which take only partial advantage of coalescent theory, on missing genotype imputation. This is validated on the human and the cattle datasets, in Tables 2 and 3 and Figures 1 and 2. For the same reason, fastPHASE is *not* expected to do well on missing haplotype allele imputation. However, from the imputation results on the mouse datasets (Table 4 and Figure 3), we see that fastPHASE is one of the best methods. Our methods NN and WNN performed very competitively against fastPHASE, sometimes better sometimes worse but overall there was no significant difference. Given that fastPHASE ran much slower than our methods, for missing haplotype allele imputation, we recommend our methods NN and WNN strongly.

### The effects of missing rate

From Figures 1, 2 and 3, we see that except the strange behavior of SVM on the high density mouse datasets, in general a higher missing rate makes the imputation more difficult. This is a non-surprising result since a higher missing rate means less deterministic data used in the imputation process. Nevertheless, as long as the missing rate stays low, such as lower than 10%, the variance is small and within ∼1*%*range. For the large jump by SVM, which was consistently there despite we simulated many more datasets and ran SVM multiple times on each, we do not have concluding insights but suspect that it is dataset dependent.

### The effects of genetic distance threshold

**Figure 4:**
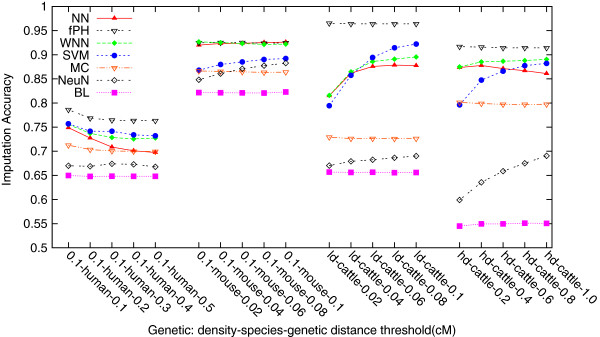
**This figure shows that the average imputation accuracy of most of the local imputation methods was not affected much by the genetic distance threshold.** For the medium density human, medium density mouse, high density cattle, and low density cattle datasets with missing rate 0.5*%*, the average imputation accuracies of most methods are nearly flat across all five thresholds, respectively, except SVM and NeuralNet were in favor of larger genetic distance thresholds on the two cattle datasets.

As we have shown earlier in missing haplotype allele imputation (Table 4 and Figure 3) that by using genetic distance thresholds, our methods NN and WNN outperformed significantly Npute, which uses a fixed number of surrounding SNP loci. On each simulated dataset, we have actually tested five different thresholds, and found that the achieved imputation accuracies do not change much. Figure 4 plots the average imputation accuracies, each over the associated 10 simulated datasets, of the imputation methods on the density-0.1 human, the density-0.1 mouse, the high-density cattle and the low density cattle datasets with missing rate 0.5%, respectively. The average imputation accuracies of most methods are nearly flat across all five thresholds, respectively, except SVM and NeuralNet were in favor of larger genetic distance thresholds on the two cattle datasets. We thus conclude that, in practice, one may choose any reasonable genetic distance threshold for imputation, as long as there are a minimum of 3 SNP loci from each side of the target SNP locus.

### Weighting the SNP loci, or not

In our NN method and Npute, when calculating sample distances, each SNP locus within the covering window contributes equally. Inspired by the Mendelian inheritance rule, SNPs closer to the target locus certainly recombine less frequently, and thus are weighted heavier in our method WNN, as the reciprocal of the genetic distances to the target locus. A bit surprising observation is that such a biologically meaningful weighting scheme did not always work out well. In more details, WNN performed no differently from NN across all datasets, with any missing rate, except on low density human datasets; nevertheless, on low density human datasets, all the six methods did not seem to perform better than BaseLine anyway. We had tested several other variations of weighting scheme including using the linakge disequilibrium *r*^2^ (obtained from the HapMap project, file name “ http://ld_chr17_ASW.txt.gz”), but none seemed to work out well. We leave such a contradictory phenomenon for future investigation.

### An alternative genotype encoding scheme

Besides the orthogonal genotype encoding scheme {(0,0,1), (0,1,0), (1,0,0), (0,0,0)}, see Methods, we had also used an *additive* scheme, {0, 1, 2, ?}, interpreting the number of minor (or any fixed) alleles in the genotype, with ? indicating a missing value. This scheme has been used in the literature, and is considered adequate when imputation is done by regression. In our experiments, the orthogonal encoding scheme showed slightly better, as our imputation is viewed as a classification task.

### Other imputation measures

We use accuracy, the proportion of correctly imputed genotypes, to measure the imputation performance. Several previous works adopt the same performance measure, but in the form of *error rate* — the proportion of incorrectly imputed genotypes [8]. For imputation by regression under the additive encoding scheme, some works use the *Pearson correlationR*^2^ as the performance measure [19]. In this work, we do not calculate *R*^2^ since we do not have “*partially*” correct imputation.

### Other imputation methods

In the literature, there are a number of other missing genotype imputation methods which are not compared against in this paper. Yu and Schaid [19] reviewed a number of direct imputation methods and compared them to fastPHASE on masked HapMap data. They found that fastPHASE provided better results, which is largely consistent to our results. The other the most promising haplotyping-based methods than fastPHASE, including MaCH [21], Impute [6] and Beagle [5], were reviewed in [23] and they perfrom comparably well to each other in terms of both efficiency and accuracy; though Beagle was shown faster and more accurate than fastPHASE on *very large* datasets [5]. For all these reasons, we chose to compare our methods NN and WNN against only fastPHASE, as the representative of the most promising haplotyping-based methods, and Npute, as the representative of the most recent direct imputation methods.

## Methods

We deal with bi-allelic SNPs, and use 0 and 1 to denote the two distinct alleles at each SNP site. We use ? to indicate a missing allele. Since it is very rare for a genotype value 0? or 1? to pass the quality control, each SNP will have one of the following four values 00, 11, 01, and ??, *i.e.* two homozygous, one heterozygous, and missing. A genotype dataset of *n* SNP loci and *m* samples is represented as an *n* × *m* matrix *M*, in which *M*(*i, j*) records the genotype of sample *j* at locus *i*. Aside from this matrix, we assume a genetic map which records the genetic distance for each locus from the start of the corresponding chromosome. When the genetic map is absent, we adopt a rough mapping of one million basepairs per centi-Morgan (cM).

In the following, our target missing value is M(i, j) = ??. We further assume that every other sample has a known value at locus *i*, for otherwise it is excluded from the imputation but to be imputed in exactly the same way as sample *j*. All our proposed imputation methods in the following perform local imputation, in the sense that only genotype values at nearby SNP loci are used in the inference of M(i, j). Here “nearby” is characterized as a window centering at locus *i*, whose radius is set to a fixed genetic distance threshold *δ*. In other words, only SNP loci within distance *δ*to the target locus *i* are used in the inference. For ease of presentation, suppose they are loci *i - L*, *i - L* + 1, …, *i* - 1, *i* + 1, *i* + 2, …, *i + R*. We will later present the range of *δ*.

### The genotype encoding scheme

We treat the imputation as a classification problem in which the three genotype values represent three classes. To this goal, we use the *orthogonal* encoding, where (0, 0, 1), (0, 1, 0), and (1, 0, 0) represent the three known genotype values respectively and (0, 0, 0) indicates a missing value. Such an encoding scheme eliminates the correlation between any two genotype values. Consequently, every missing genotype is either imputed correctly or wrongly, but never *partially* correct.

### One nearest neighbor (NN) and its weighted variant (WNN)

In the one nearest neighbor method, the key issue is to define the distance between a training sample and the target sample, both represented as 3(*L + R*)-dimensional binary vectors. We adopted the Hamming distance. Such a distance function differs slightly from the one used in Npute, where the entry distance between two missing values is set to 1 [18] — while we set it to 0.

After all the distances are calculated, the classical one nearest neighbor method selects a closest training sample to the target for inference. We do slightly differently in our NN method, to use all training samples that are the closest to the target sample. By checking the known genotype values at SNP locus *i* for all these closest neighbors, the missing value *M*(*i, j*) is imputed as the majority genotype; when there are ties, our NN method continues to recruit the training samples that are the second closest to the target, then the sample majority vote applies, and so on. In the worst case where all training samples are used for inference, we break ties arbitrarily.

In the weighted variant WNN, the weight of SNP locus *i*’ in the covering window is set to the reciprocal of the genetic distance from locus *i*’ to *i*, and that all the *L* + *R* weights are scaled into the range [0, 100]. In fact, multiple ranges have been tested in our preliminary experiments, and [0, 100] is chosen for its the most stable performance. Afterwards, the distance between two samples are analogously calculated, so does the modified majority voting scheme.

### Support vector machine (SVM)

Support vector machines (SVMs) are a useful algorithm for classification tasks. We use the SVM software package LIBSVM [27] for our genotype imputation.

Similar as in NN, each training sample *k* (*k* ≠ *j*) is represented as a 3(*L + R*) + 1-dimensional binary vector (**x**_*k*_, *y*_*k*_), in which *y*_*k*_ ∈ {0,1,2} is the genotype value (or the class label) at locus *i*. The target sample *j* is also represented as a 3(*L + R*) + 1-dimensional binary vector, with its *y*_*j*_value to be settled. The support vector machine is a minimization problem on the training dataset [28]:

(1) $$\begin{array}{cc} \underset{\alpha }{\text{minimize}}\; & \frac{1}{2}\alpha^{⊤}Q\alpha -e^{⊤}\alpha \\ \text{subject to}\; & y^{⊤}\alpha =0,\\ 0\leq \alpha_{k}\leq c,\;k=1,2,\ldots ,\\ j-1,j+1,j+2,\ldots ,m, \end{array}$$

where *c* > 0 is the penalty term of the errors, **e** = ^(1,1,…,1)⊤^ is an (*m* - 1)-dimensional vector, **y**= ${\left(y_{1},y_{2},\ldots ,y_{j-1},y_{j+1},y_{j+2},\ldots ,y_{m}\right)}^{⊤}$ is the (*m* - 1)-dimensional class label vector, *Q*_*ℓk*_ =*y*_*ℓ*_*y*_*k*_*K*(**x**_*ℓ*_**x**_*k*_), and $K(x_{\ell },x_{k})=\phi {\left(x_{\ell }\right)}^{⊤}\phi (x_{k})$ is the kernel function. For each training sample **x**_*k*_, function *ϕ* maps it into a higher dimensional space. SVM can be viewed as a minimization problem that tries to find a hyperplane with maximal margin in this higher dimensional space. In our implementation, we adopt the radial basis function (RBF) in which $K(x_{\ell },x_{k})=\text{exp}\;(-\gamma ||x_{\ell }-x_{k}|{|}^{2})$ and *γ* > 0. It has been reported that the RBF kernel is able to handle the case where the relation between the class label and the attributes is nonlinear. The target sample class label *y*_*j*_ is calculated by fitting its attribute vector **x**_*j*_ into the trained model.

As seen from the above, there are two parameters to be learned for the SVM with an RBF kernel, *c* and *γ*, for which no known best values exist for our imputation problem. We used a 10-fold cross-validation to search for their values out of a pre-defined grid {2^−5^, 2^−3^, …, 2^15^} × {2^−15^, 2^−13^,…, 2^3^} [27]. Note that we do not try to achieve a high training accuracy on the training dataset but rather to find a model that is general enough to work well for real imputation. In the 10-fold cross-validation, the training samples are divided into 10 subsets of equal size, each subset is tested using the classifier trained on the remaining 9 subsets. The cross-validation accuracy is the percentage of samples which are correctly classified.

### Neural network (NeuralNet)

We employ a standard three-layer feed-forward network with a gradient descent training algorithm [29]. The sample representation **x**_*k*_is the same as in SVM, except that the class label is no longer a single digit but a three-dimensional vector **y**_*k*_∈ {(1, 0, 0), (0, 1, 0), (0, 0, 1)}. Consequently, our neural network has exactly 3(*L* + *R*) input neurons and 3 output neurons. The number of neurons in the hidden layer is *M*, which is set to *L* + *R* in our implementation.

Let *W* = {***α***_0_, ***α***_1_, ***α***_2_, …,***α***_*M*_, ***β***_0_, ***β***_1_, ***β***_2_, ***β***_3_} denote the set of network weights to be trained, where ***α***_0_, ***β***_1_, ***β***_2_, and ***β***_3_ are *M*-dimensional vectors, ***α***_*m*_ is a 3(*L* + *R*)-dimensional vector for *m* = 1, 2, …, *M*, and ***β***_0_ = (*β*_01_, *β*_02_,*β*_03_) is a 3-dimensional vector. For each training sample **x**_*k*_, we have

(2) $$\begin{array}{cc} h_{\text{km}} & =\alpha_{0m}+\alpha_{m}^{⊤}x_{k},\;m=1,2,\ldots ,M;\\ z_{\text{km}}=\sigma & (h_{\text{km}})=\frac{1}{1+\text{exp}\;(-h_{\text{km}})},\;m=1,2,\ldots ,M;\\ t_{\mathrm{k\ell}}=\beta_{0\ell }+\beta_{\ell }^{⊤}z_{k},\;\ell =1,2,3. \end{array}$$

In the above, *σ* (·) is the *softmax* function. The training phase is to minimize the error function

(3) $$E(W)=\underset{k\neq j}{\sum }\overset{3}{\underset{\ell =1}{\sum }}(-t_{\mathrm{k\ell}}\log(y_{\mathrm{k\ell}})),$$

for which we use a batch version of the gradient descent algorithm starting with a random guess of *W *, a learning rate *η*, and a momentum *μ* all in the range [0, 1]. The training process is iterated for 20 times to avoid over-fitting. Afterwards, the class label vector of the target sample **y**_*j*_ is calculated by fitting its attribute vector **x**_*j*_ into the network. The final class label is set by checking which one of the three entries has the largest value, with ties broken arbitrarily.

### First order Markov chains (MC)

Markov chains are also able to capture sequential dependencies. We build two first order local Markov chains (MC) using the *L* up-stream SNPs and the *R* down-stream SNPs inside the covering window, respectively, together with locus *i*, to predict the most likely genotype values for *M*(*i, j*), and then combine them to vote for the final value.

In the up-stream MC, the initial genotype probability distribution is for locus *i-L*, and the transition probabilities from locus *k* to *k* + 1, for *k*=*i*−*L*,*i*−*L* + 1,…,*i*−1, are taken as the conditional probabilities *P*(*X*_*k* + 1_| *X*_*k*_). A care is paid to the place where some genotype frequency at a locus is 0 counted from the training samples, for which we use an add-one smoothing scheme to lift it to a non-0 value. During the imputation phase, in case of a missing value in the target sample *j*, we enumerate all possible genotype values using their frequencies in the training samples. At the end, for each genotype value, we obtain a probability of occurrence from the up-stream MC. Analogously, another probability is obtained from the down-stream MC. The product is taken as the likelihood of this genotype value at locus *i*, and the final value is set to the one with the largest likelihood.

## Conclusions

We have presented fast and accurate missing SNP genotype and missing haplotype allele imputation methods NN and WNN, based on the Mendelian inheritance rule but to avoid the complex and time-consuming phasing step. Our methods are local, such that only the SNPs within a short genetic distance vicinity are taken into the imputation process. Extensive simulation experiments showed that our methods performed very well compared to previously the best methods, fastPHASE for missing genotype imputation and Npute for missing haplotype allele imputation. Our methods lost to fastPHASE slightly on missing genotype imputation, but competitively well on missing haplotype allele imputation. In terms of imputation time, our methods outperformed fastPHASE several orders of magnitude.

We have applied our NN method to impute the ASW human genotype dataset and the Perlegen mouse dataset. The imputed datasets, the simulated datasets, more detailed imputation result statistics, and source codes of our implemented imputation methods (NN, WNN, SVM, MC, NeuralNet, BaseLine) are available in the supplementary materials [26].

## Competing interests

The authors declare that they have no financial or non-financial competing interests.

## Authors’ contributions

YW and ZC carried out most of the computational experiments and drafted the Methods section of the manuscript. PS, SM, RG and GL participated in the discussion and suggested datasets for simulation studies. GL participated in the design of the study and its coordination, and drafted the manuscript. All authors read and approved the final manuscript.
